# A Rapid, Reliable RP-UPLC Method for Large-Scale Analysis of Wheat HMW-GS Alleles

**DOI:** 10.3390/molecules26206174

**Published:** 2021-10-13

**Authors:** Su-Bin Lee, Yu-Jeong Yang, Sun-Hyung Lim, Yong Q. Gu, Jong-Yeol Lee

**Affiliations:** 1 National Institute of Agricultural Science, RDA, Jeonju 54874, Korea; lsb36712@korea.kr (S.-B.L.); yujinge@korea.kr (Y.-J.Y.); 2 Division of Horticultural Biotechnology, Hankyong National University, Anseong 17579, Korea; limsh2@hknu.ac.kr; 3 USDA-ARS, Western Regional Research Center, 800 Buchanan Street, Albany, CA 94710, USA; yong.gu@usda.gov

**Keywords:** HMW-GS, RP-UPLC, allelic analysis, large wheat germplasm

## Abstract

High-molecular-weight glutenin subunits (HMW-GS) account for only 10% of total wheat storage proteins, but play an important role in the processing quality of wheat flour. Therefore, identifying HMW-GS alleles associated with good end-use quality provides important information for wheat breeders. To rapidly, accurately and reproducibly identify HMW-GS, we established an optimized reversed-phase ultra-performance liquid chromatography (RP-UPLC) method. Separation parameters were optimized using an ACQUITY UPLC Protein BEH C_4_ column and stepwise ACN gradient, and the separation patterns and retention times (RTs) of 22 subunits were comparatively analyzed in 16 standard wheat cultivars. All HMW-GS proteins were well separated within about 5.5 min, and all analyses were complete within 12 min. We distinguished the 16 subunits based on RT, although three subunits in 1Bx (1Bx7/1Bx7^OE^ and 1Bx17) and three subunits in 1By (1By8*, 1By9 and 1By15) had overlapping RTs; these were differentiated by SDS-PAGE. To distinguish 1Bx7 and 1Bx7^OE^, which differ in protein abundance, RP-UPLC was combined with PCR analysis of DNA junction markers. The optimized method was successfully applied to determine HMW-GS alleles in a large collection of bread wheat germplasm (1787 lines). This protocol is an appropriate option for selecting lines harboring favorable HMW-GS alleles in wheat breeding.

## 1. Introduction

Gluten, composed of polymeric glutenin and monomeric gliadin, is the primary factor that produces visco-elasticity in wheat dough and is important for wheat end-use quality [1,2]. Based on their molecular weights and primary structures, glutenins are divided into high-molecular-weight (HMW-GS, MW = 67–100 kDa) and low-molecular-weight (LMW-GS, MW = 32–35 kDa) glutenin subunits [3,4,5]. Each *Glu-1* locus contains two linked genes that encode x-type and y-type HMW-GS. The x-type subunits generally have a higher molecular weight than the y-type subunits. In general, bread wheat contains three to five full length genes expressing HMW-GS, as *Glu-A1* encodes an x-type subunit or is absent, *Glu-B1* may encode both x- and y-type or only x-type HMW-GS and *Glu-D1* encodes both types [1,6,7,8]. HMW-GS are crucial to dough quality because they form large polymeric backbones through disulfide bonds. This polymeric structure influences the rheological properties of dough and HMW-GS allelic variation is thought to account for 47–60% of wheat bread-making quality [1,6,9]. Therefore, the qualitative and quantitative effects of individual subunits are important for dough properties and bread-making quality [10].

The association between specific HMW-GS and bread-making quality has been extensively studied and can be quantified as the *Glu-1* quality score, a numeric scale used in wheat breeding [11,12]. The *Glu-1* quality score is defined as the sum of the contributions of all individual subunits from the *Glu-A1*, *Glu-B1* and *Glu-D1* loci in a specific wheat line. For example, at *Glu-A1*, 1Ax1 and 1Ax2* contribute to a higher quality score than the null allele [1,7]. At *Glu-B1*, the subunit combination 1Bx17 + 1By18 results in higher dough strength than 1Bx20 + 1By20 [1,13]. Moreover, overexpression of 1Bx7 (1Bx7^OE^), which has two functional copies of 1Bx7genes, increases dough strength [14,15,16,17]. *Glu-D1* is known to have a greater impact on bread-making quality than *Glu-A1* and *Glu-B1*. In particular, 1Dx5 + 1Dy10 is associated with superior end-use quality [1,7].

To determine the HMW-GS allelic composition of bread wheat, techniques such as sodium dodecyl sulfate-polyacrylamide gel electrophoresis (SDS-PAGE), polymerase chain reaction (PCR) analysis and reversed-phase high-performance liquid chromatography (RP-HPLC) are commonly and widely used as alternatives to techniques requiring expensive instruments, such as high-performance capillary electrophoresis (HPCE) or matrix-assisted laser desorption/ionization time-of-flight mass spectrometry (MALDI-TOF-MS) [18,19,20,21,22,23,24,25,26]. Each of these three methods has drawbacks, however. Since SDS-PAGE distinguishes proteins by their differences in electrophoretic mobility, it is sometimes inadequate to distinguish subunits with similar molecular weights, resulting in incorrect identification. PCR analysis is simple and is commonly used in many breeding programs, but does not cover all HMW-GS allelic variations present in hexaploid wheat. Although RP-HPLC is fully automated, shows high reproducibility and provides the possibility for quantitative analysis, it also tends to have longer separation times and consumes larger amounts of reagents (such as solvents) than other methods [23].

Rapid and accurate identification of HMW-GS during a breeding program is of paramount importance for wheat quality improvement. In this study, we developed an optimized RP-UPLC method to identify these molecules by adopting a recently developed column to separate high-molecular-weight proteins and using stepwise gradient conditions to markedly shorten the time and increase resolution for bread wheat samples. With this optimized method and auxiliary methods, we successfully identified HMW-GS alleles of a large, worldwide collection of 1787 wheat lines, thus providing valuable resources for integrating wheat end-use quality data into breeding programs in the future.

## 2. Results and Discussion

### 2.1. Optimization of RP-UPLC Conditions

**Figure 1:**
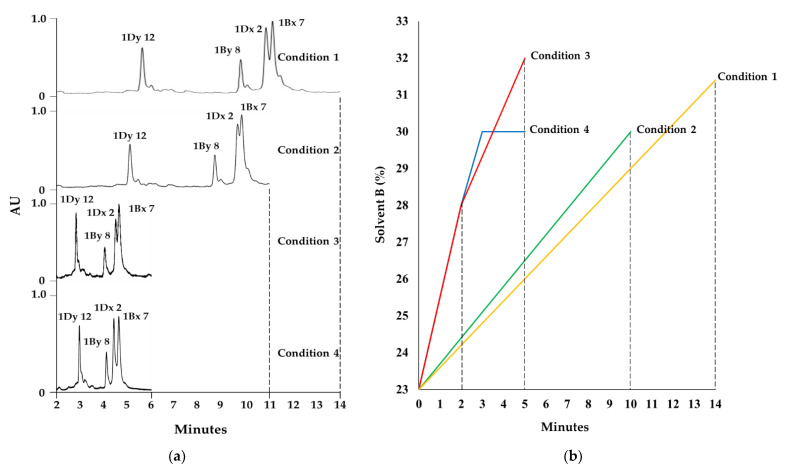
Optimization of RP-UPLC separation by changing ACN gradient conditions according to two linear gradients (Conditions 1 and 2) or two stepwise gradients (Conditions 3 and 4) using the same type of ACQUITY Protein BEH 300 C_4_ column on samples from the Chinese Spring wheat cultivar. (**a**) Comparison of chromatogram patterns. (**b**) Detailed ACN gradient parameters in each condition.

UPLC increases separation efficiency and provides superior resolution and sensitivity compared to classic HPLC because of its much wider range of linear velocities and its higher flow rates and back-pressures. Here, we optimized the RP-UPLC method for HMW-GS analysis by testing two columns and four mobile-phase gradient conditions to reduce the analysis time while increasing the resolution. Specifically, the ACQUITY UPLC peptide BEH C_18_ column (particle size 1.7 µm, 2.1 mm × 100 mm id, 300 Å) and ACQUITY UPLC Protein BEH C_4_ column (particle size 1.7 µm, 2.1 mm × 100 mm id, 300 Å), which are frequently used for peptide or protein separation, were tested. The same elution conditions were used for the glutenin fraction extracted from the reference cultivar Chinese Spring (CS), consisting of a column temperature of 60 ℃ and a flow rate of 0.4 mL/min with an acetonitrile (ACN) linear gradient from 23% to 41% for 30 min. Using the C_4_ column resulted in a faster elution time than the C_18_ column (Figure S1), prompting us to select the C_4_ column for further experiments. Column temperature and flow rate, which are critical separation parameters, showed good resolution at 60 °C and 0.4 mL/min, respectively, when compared across several conditions (data not shown), and therefore this temperature and flow rate were used for later experiments. To establish analysis conditions for the mobile phase, two linear gradient conditions (Condition 1: ACN 23–41% for 30 min; Condition 2: ACN 23–30% for 10 min) and two stepwise gradient conditions (Condition 3: ACN 23–28% for 2 min, 28–32% for 3 min; Condition 4: ACN 23–28% for 2 min, 28–30% for 1 min and 30% for 2 min) were tested. The resulting chromatograms are shown in Figure 1. Under Condition 4, all subunits of HMW-GS in CS were effectively resolved, and all peaks eluted within about 5 min, thus substantially reducing the analysis time.

To check the reproducibility of this optimized method, we performed the same analysis of the HMW-GS complement from the CS ten times under the same analytic condition and then calculated the average retention times (RTs), peak areas and relative standard deviations (RSD%) for RTs for the four subunits. The RSD% of RTs and peak areas varied by less than 0.21% and 1.18%, respectively, across technical replicates, demonstrating the high reproducibility of this method (Figure S2 and Table S1).

**Figure 2:**
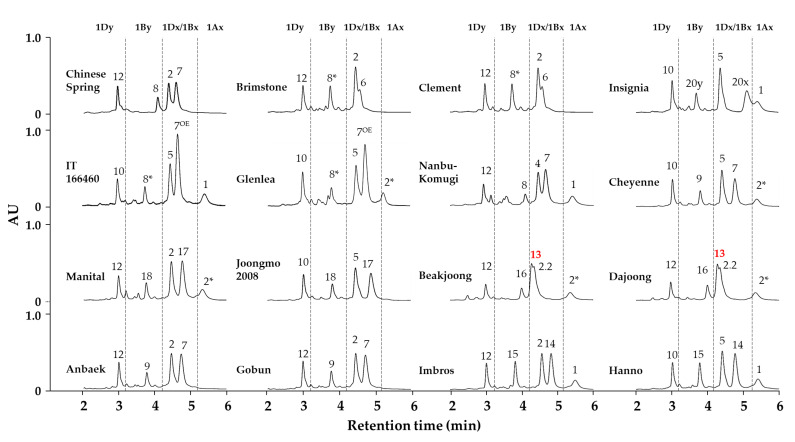
RP-UPLC chromatograms of HMW-GS in 16 standard wheat cultivars. The HMW-GS identified in each cultivar are indicated above the corresponding peaks. The single exception to the elution order, 1Bx13, is indicated in red.

**Figure 3:**
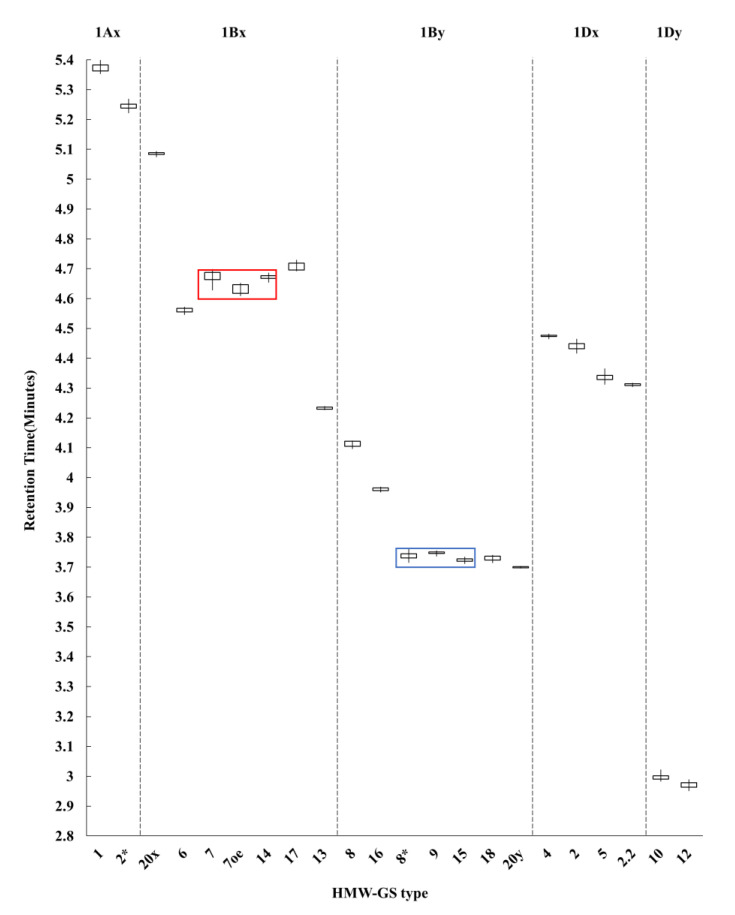
Box and whisker plot of retention times for 22 HMW-GS in 16 standard wheat cultivars. The red and blue boxes indicate subunits with overlapping retention times that cannot be distinguished from the other subunits on the basis of retention time alone. 1Bx7/1Bx7^OE^ and 1Bx14 in the red box and 1By8*, 1By9 and 1By15 in the blue box were identified by SDS-PAGE.

The ACQUITY UPLC Protein BEH (Ethylene Bridged Hybrid) C_4_ column was designed to separate large molecular weight proteins and has shown excellent performance in separating many challenging proteins, overcoming the shortcomings of 100% silica-based materials [27,28,29,30,31]. Among the glutenin fraction, HMW-GS are more hydrophilic and larger than LMW-GS. Using the ACQUITY UPLC Protein BEH C_4_ column therefore allowed us to shorten elution time while retaining good separation. To further reduce analysis time as in Condition 4, two short gradients, such as 0–2 min ACN 23–28% and 2–3 min ACN 28–30%, rather than the 0–3 min ACN 23–30% gradient of Condition 3, were applied. Under this condition, the subunits 1Dy12 and 1Dy10 (Figure 2 and Figure 3, Table 1 and Table 2,) were successfully separated, which was not achieved in a previous study [25]. In addition, the separation of 1Dx and 1Bx improved as compared to Condition 3 due to the isocratic conditions of using 30% ACN for 3–5 min. These stepwise gradient conditions are an important reason for the observed combination of improved resolution and reduced analysis time.

### 2.2. Identification of HMW-GS Compositions in Standard Wheat Cultivars

In this study, we identified the composition for 22 HMW-GS in 16 standard wheat cultivars, all reported in various earlier publications, using our optimized RP-UPLC method along with auxiliary methods (Table 1, Figure 2 and Figure 3, Figures S3 and S4). The 22 subunits were: two of the 1Ax type (1Ax1 and 1Ax2*), seven of the 1Bx type (1Bx6, 1Bx7, 1Bx7^OE^, 1Bx13, 1Bx14, 1Bx17 and 1Bx20), seven of the 1By type (1By8, 1By8*, 1By9, 1By15, 1By16, 1By18 and 1By20), four of the 1Dx type (1Dx2, 1Dx2.2, 1Dx4 and 1Dx5) and two of the 1Dy type (1Dy10 and 1D12). With the exception of the subunits 1Dx20 and 1Dy20 of cultivar Insignia, each remaining subunit was represented by at least two standard cultivars to improve the accuracy of the analysis (Table 1). All wheat cultivars were analyzed ten times, and the resulting average RTs and RSD% of each subunit are reported in Table 2. The RSD% of RTs were less than 0.42%, illustrating high reproducibility of the optimized method. 

To determine whether each subunit can be distinguished based on retention time alone, the RT of each 22 HMW-GS was plotted in a box-and-whisker plot (Figure 3). 1Dy12, the most hydrophilic subunit, eluted at 2.970 min and 1Ax1, the most hydrophobic subunit, eluted at 5.373 min. Most HMW-GS eluted in the order (from fastest to slowest RT) 1Dy < 1By < 1Dx < 1Bx < 1Ax, with the exception of 1Bx13. In the cultivars Baekjoong and Dajoong, HMW-GS eluted in the order 1Dy12 < 1By16 < 1Bx13 < 1Dx2.2 < 1Ax2* (Figure 2 and Figure 3). The two subunits encoded by the *Glu-A1* locus, 1Ax1 and 1Ax2*, showed the greatest hydrophobicity among all HMW-GS, and they eluted last, making them easily distinguishable by RT, as shown in Figure 3.

Of the subunits encoded by the *Glu-B1* locus, 1Bx subunits eluted more slowly than 1By subunits. The 1Bx6, 1Bx13, 1Bx17 and 1Bx20 subunits could generally be distinguished, but not in the case of 1Bx7/1Bx7^OE^ and 1Bx14 due to their overlapping RTs. However, 1Bx7/1Bx7^OE^ and 1Bx14 were easily distinguishable by SDS-PAGE analysis based on their distinct electrophoretic mobility (Figure S3). The 1Bx7^OE^ subunit typically exhibits higher abundance resulting from a duplication of the 1Bx7 gene. The 1Bx7^OE^ and 1Bx7 subunits were also distinguishable by PCR amplification using left and right junction primers between the duplicated segments and long terminal repeat (LTR) retrotransposon borders that gave rise to 1Bx7^OE^ [5,17]. Therefore, the cultivars presumed to carry 1Bx7 or 1Bx7^OE^ based on measured RT during RP-UPLC analysis were genotyped for 1Bx7^OE^ (Figure S4). The higher accumulation of subunit 1Bx7 in cultivars harboring 1Bx7^OE^ increases dough strength and is considered the main reason for its large positive effect on bread-making quality [15,16,32,38]. Of the 1By subunits, 1By8, 1By16 and 1By20 were distinguishable based on their RT in RP-UPLC, while 1By8*, 1By9, 1By15 and 1By18 were not. However, the presence of the 1By18 can be inferred from the presence of the linked pair 1Bx17 (1Bx17 + 1By18), as seen in Manital and Joongmo (2008). The remaining 1By8*, 1By9 and 1By15 subunits were distinguishable only through SDS-PAGE analysis (Figure S3).

For the *Glu-D1* locus, all the x-type (1Dx4, 1Dx5, 1Dx2 and 1Dx2.2) and y-type (1Dy10 and 1Dy12) subunits were distinguishable based on their non-overlapping RTs. In practical breeding programs for wheat quality improvement, it is also important to identify heterozygotes (e.g., 2 + 12/5 + 10) among breeding lines. For this purpose, we extracted and mixed the two HMW-GS fractions from two varieties (Insignia and Manital) and RP-UPLC analyses were performed to test our optimized method. As a result, it was possible to identify subunits except for 1Dy 10 + 12 and 1Ax 1 + 2*, derived from these varieties (Figure S5). The two mixture peaks could be inferred as 10 + 12 and 1 or/and 2* by linked pairs or retention time.

Yan et al. [23] reported a rapid RP-UPLC method showing good separation patterns and high reproducibility for the identification of HMW-GS. Their method adopted an ACQUITY UPLC BEH 300 C_18_ column (particle size 1.7 µm, 2.1 mm × 50 mm id, 300 Å) using the Waters ACQUITY UPLC system with separation parameters consisting of an ACN linear gradient of 21–47% for 30 min, a flow rate of 0.55 mL/min and a column temperature of 55 ℃. With their method, all HMW-GS eluted in about 12 min, resulting in good separation of 34 subunits from 111 wheat cultivars and wheat relatives, but the total analysis time was more than 30 min. The optimized RP-UPLC method presented here used a column designed for large proteins combined with innovative stepwise ACN gradients to significantly reduce HMW-GS elution time to about 5.5 min, for a total analysis time of about 12 min. Among the 22 subunits, six HMW-GS with overlapping RTs or whose encoding genes were not linked to other HMW-GS pairs were identified by SDS-PAGE and PCR analysis to prevent potential misleading results when the RP-UPLC method was used alone, thus increasing the reliability of the analysis.

### 2.3. Applying the Optimized Method to a Large Collection of Wheat Germplasm

The optimized method established in this study was then used to determine the HMW-GS complement in a large-scale wheat genetic resource consisting of 1787 wheat lines from the National Agrobiodiversity Center in Korea. The 22 subunits from the 16 standard wheat cultivars tested above were used as external standards for HMW-GS allelic analysis. The results of this HMW-GS allelic analysis are listed in Table S2. This germplasm collection comprised wheat cultivars, breeding lines and collected accessions from South and North Korea and around the world. All wheat samples in the study were characterized by the presence of three to five subunits, which is a typical HMW-GS profile for hexaploid wheat (Table S2).

The frequencies of 27 HMW-GS alleles at *Glu-1* were determined for all 1787 wheat lines (Table 3). At *Glu-A1*, we obtained frequencies of 23.29%, 43.31% and 33.30% for the three alleles 1Ax1, 1Ax2* and null, respectively. *Glu-B1* presented the largest number of alleles (17) among *Glu-1* loci, with 1Bx7 + 1By9 (28.15%), 1Bx7 + 1By8 (26.47%), 1Bx7 + 1By8* (12.26%), 1Bx17 + 1By18 (12.26%) and 1Bx20 + 1By20 (6.72%), five alleles, accounting for 85.86% of all *Glu-B1* alleles. Allelic pairs were identified at *Glu-B1* with 1Bx7^OE^ known to be beneficial for bread-making quality: 1Bx7^OE^ + 1By8* and 1Bx7^OE^ + 1By9, in 33 (1.85%) and two (0.11%) accessions, respectively. We determined the genotype at 1Bx7^OE^ by PCR amplification with left and right junction primers for germplasm in which 1Bx7 and 1Bx7^OE^ could not be separated by RP-UPLC analysis (Figure S4). The 1Bx7^OE^ + 1By9 pair was described in Zheng et al. [39] in one out of 485 common wheat landraces collected from the Yangtze River basin in China. In this study, we identified two (line 504 and 620) lines from the collection of 1787 lines, both of which were bread wheat varieties from Mexico. At *Glu-D1*, three pairs [1Dx5 + 1Dy10 (46.84%), 1Dx2 + 1Dy12 (42.42%) and 1Dx2.2 + 1Dy12 (8.39%)] among the seven alleles accounted for 97.65% of the lines present in the germplasm collection.

**Figure 4:**
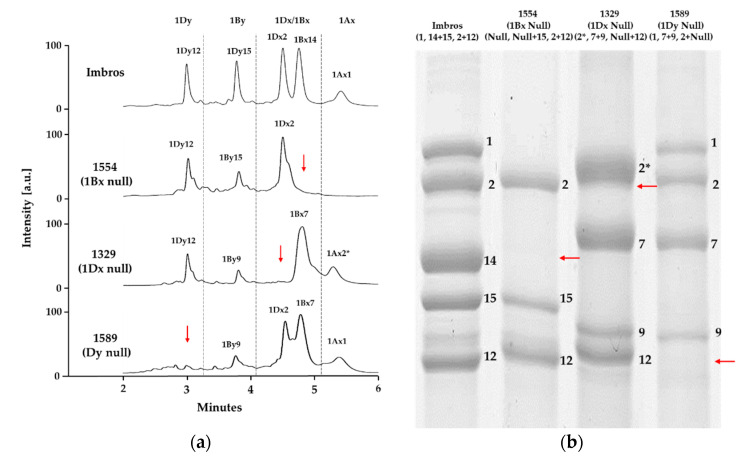
Representative RP-UPLC chromatograms (**a**) and SDS-PAGE profiles (**b**) of 1Bx, 1Dx and 1By null wheat lines. The regions lacking the respective HMW-GS are indicated by red arrows.

Our analysis also allowed the identification of wheat lines with unique null allelic combinations. The *Glu-B1* locus commonly harbors genes encoding 1Bx + 1By or only 1Bx alleles, but we detected 12 1Bx nulls (one with 1By8, one with 1By8*, one with 1By9, seven with 1By15 and two with 1By20). Similarly, 1Dx + 1Dy alleles are typical at *Glu-D1*, but we identified one 1Dx null (with 1Dy12) and three 1Dy nulls (one with 1Dx2, two with 1Dx5) (Figure 4, Table S2).

The 1787 accessions represented 95 possible allelic combinations (Table S3). Allelic combinations associated with good bread-making quality that should be included at each locus were: either 1Ax1 or 1Ax2* at *Glu-A1*; any one of the pairs 1Bx7 + 1By8, 1Bx17 + 1By18, 1Bx7^OE^ + 1By8* and 1Bx7^OE^ + 1By9 at *Glu-B1*, and 1Dx5 + 1Dy10 at *Glu-D1*, which corresponded to 248 lines, accounting for 13.88% of the total.

RP-UPLC is a relatively easy-to-use technique that has recently been widely implemented. Although our method showed a short processing time per sample, one limitation is that samples will be run one by one with a single machine, while multiple samples can be run simultaneously on a single SDS page gel. The optimized RP-UPLC method presented here is a powerful and reliable method to determine HMW-GS composition in breeding programs for wheat quality improvement. In this HMW-GS analysis of large-scale genetic resources, we highlighted wheat lines with allelic combinations conveying good bread-making quality that can be directly included in breeding programs for quality improvement.

## 3. Materials and Methods

### 3.1. Plant Materials

Seeds for sixteen standard bread wheat (*Triticum aestivum* L.) cultivars were kindly provided by the U.S. National Plant Germplasm System and the National Institute of Crop Science (NICS, Jeonju, Korea), as listed in Table S2. Seeds for 1787 hexaploid wheat lines were obtained from the National Agrobiodiversity Center (Jeonju, Korea) and grown at NICS in 2017 as reported in Jang et al. [26]. All grain samples used in the experiment were crushed with a cyclone sample mill (Udy Corporation, Fort Collins, CO, USA), and were turned into flour.

### 3.2. Glutenin Extraction and Precipitation 

The glutenin fraction was extracted from wheat flour according to Singh et al. [40] with minor modifications. To remove monomeric gliadins, 1 mL of 50% (*v*/*v*) propanol was added to 50 mg of wheat flour, incubated at 65 °C for 30 min and centrifuged at 10,000× *g* for 10 min, after which the supernatant was discarded. This step was repeated twice. To extract polymeric glutenin, 500 µL of extraction buffer (50% (*v*/*v*) propanol, 80 mM Tris-HCl pH 8.0 containing 1% (*w*/*v*) dithiothreitol (DTT)) was added to the above pellet, incubated at 65 °C for 30 min and centrifuged at 10,000× *g* for 5 min. Subsequently, another 500 µL of extraction buffer containing 1.4% (*v*/*v*) 4-vinylpyridine instead of 1% DTT was added and the sample was incubated at 65 °C for 15 min for glutenin alkylation. After centrifugation at 10,000× *g* for 5 min, the supernatant was transferred to a new tube and stored at −80°C for further experiments.

Glutenin precipitation for RP-UPLC analysis was performed as described by Melas et al. [41]. The glutenin extracts were precipitated by the addition of acetone to a final concentration of 40% (*v*/*v*). Then, 600 µL of cold acetone was added to 900 µL of the collected supernatant in a new 2 mL tube and incubated at −20 °C overnight. After centrifugation at 10,000× *g* at 4 °C for 10 min, the resulting protein pellet was rinsed with 500 µL acetone and centrifuged at 4 °C for 10 min. Finally, the protein pellet was completely air-dried.

For SDS-PAGE analysis, the extracted glutenin fractions were precipitated using 15% (*v*/*v*) trichloroacetic acid (TCA)/acetone at −20 °C overnight. Then, 50 µL of the supernatant and 200 µL of cold acetone containing 15% (*v*/*v*) TCA were mixed in a 1.5 mL tube and incubated at −20 °C overnight. After centrifugation at 10,000× g at 4 °C for 10 min, the resulting protein pellet was rinsed with 200 µL of acetone containing 0.07% (*w*/*v*) DTT and centrifuged at 4 °C for 10 min. Finally, the protein pellet was completely air-dried.

### 3.3. RP-UPLC 

RP-UPLC analysis was performed on an ACQUITY UPLC H-Class System (Waters Corp, Milford, MA, USA). All mobile-phase solvents for RP-UPLC analysis were of high purity and were purchased from Fisher Scientific (USA).

To establish optimal RP-UPLC analysis conditions for HMW-GS, the ACQUITY UPLC Protein BEH C_4_ Column (Waters Corp, USA, particle size 1.7 µm, 2.1 × 100 mm id, pore size 300 Å) and ACQUITY UPLC Peptide BEH C_18_ Column (Waters Corp, USA, particle size 1.7 µm, 2.1 × 100 mm id, pore size 300 Å) were tested with the same analytical parameters. Water and acetonitrile (ACN), both containing 0.06% (*v*/*v*) trifluoroacetic acid (TFA), were used as mobile-phase solvents A and B, respectively. The dried protein pellet was completely resuspended in 200 µL of 20% (*v*/*v*) ACN containing 0.06% TFA and filtered using a 0.22 µm PVDF syringe filter (Whatman, UK). Four microliters of each sample was injected for separation. HMW-GS were eluted using a linear gradient of 23–41% solvent B for 30 min with a flow rate of 0.4 mL/min and a column temperature of 60 °C and monitored at a wavelength of 210 nm.

After determining that the ACQUITY UPLC Protein BEH C_4_ Column performed better, four mobile-phase gradient conditions were tested: two linear gradients (Condition 1: solvent B 23–41% for 30 min; Condition 2: solvent B 23–30% for 10 min) and two stepwise gradients (Condition 3: solvent B 23–28% for 0–2 min, 28–32% for 2–5 min; Condition 4: solvent B 23–28% for 0–2 min, 28–30% for 2–3 min, 30% for 3–5 min). Finally, optimal HMW-GS elution conditions were determined to be Condition 4 with a washing step (solvent B 30–90% for 30 sec, 90% for 1.5 min, 90–23% for 30 sec, 23% for 4.5 min), with the remaining analytical parameters as above.

### 3.4. SDS-PAGE 

SDS-PAGE analysis was performed on a Hoefer SE260 Mighty Small II electrophoresis unit with reference to Jang et al. [25]. Each precipitated protein pellet was resuspended in 20 µL of sample buffer (50 mM Tris-HCl pH 6.8, 2% (*v*/*v*) β-mercaptoethanol, 2% (*w*/*v*) SDS, 20% (*v*/*v*) glycerol and 0.01% (*w*/*v*) bromophenol blue) and 4 µL was loaded onto a 12.5% SDS-PAGE gel. Xpert Prestained Protein Marker (10–240 kDa, GenDEPOT, Katy, TX, USA) was used as protein marker. After electrophoresis, the gel was stained with 1% (*w*/*v*) Coomassie Brilliant Blue R-250 and destained in destaining solution (water:acetic acid:methanol (8:1:1 *v*/*v*/*v*)).

### 3.5. Genomic DNA Extraction and PCR Analysis

Genomic DNA was extracted from 50 mg of wheat flour for each wheat cultivar using the GeneAll Exgene Plant SV mini kit (GeneAll, Seoul, Korea) following the manufacturer’s instructions. The extracted genomic DNA was quantified and its quality assessed on a NanoDrop spectrophotometer (Thermo Scientific, Waltham, MA, USA), and then it was diluted to 50 ng/μL. PCR analysis to discriminate between 1Bx7 and 1Bx7^OE^ was performed using the method described by [5,17]. PCR was performed in a reaction volume of 20 μL using 150 ng of genomic DNA, 1.25 U of Go Taq DNA polymerase (Promega, USA), 1× Green Go Taq reaction buffer (containing 1.5 mM MgCl_2_), 200 μM of dNTP mix (Bioneer, Daejeon, Korea) and 10 pmol each of forward and reverse primers. Left junction primers were: forward 5′-ACGTGTCCAAGCTTTGGTTC-3′ and reverse 5′-GATTGGTGGGTGGATACAGG-3′, and right junction primers were forward 5′-CCACTTCCAAGGTGGGACTA-3′ and reverse 5′-TGCCAACACAAAAGAAGCTG-3′ [17]. Amplification conditions for PCR were an initial cycle at 95 °C for 5 min, followed by 34 cycles of 94 °C for 30 s, 57 °C for 30 s and 72 °C for 1 min, and then a final extension at 72 °C for 5.25 min. PCR products were resolved on 1.5–2.0% agarose gels in 0.5× Tris borate EDTA (TBE) buffer, stained with ethidium bromide and visualized under UV.

## 4. Conclusions

The HMW-GS composition of wheat grains has key implications for grain quality for specific end uses, with different alleles providing advantages for different uses, from noodles to bread dough. This study presented an optimized RP-UPLC method for determining the HMW-GS alleles in wheat and validated this method in a large collection of bread wheat germplasm (1787 lines). The resulting protocol provides a valuable tool for selecting lines harboring favorable HMW-GS alleles in wheat breeding, particularly when combined with auxiliary methods to identify alleles whose encoded proteins migrate with similar retention times. Future research in the area may further increase the resolution of the analysis and improve throughput.

## Figures and Tables

**Table 1:** List of 16 standard wheat cultivars used in this study for HMW-GS analysis.

Cultivar	HMW-GS			References
	*Glu-A1*	*Glu-B1*	*Glu-D1*	
Anbaek	Null	7 + 9	2 + 12	Jang et al. [5,25]
Baekjoong	2*	13 + 16	2.2 + 12	Jang et al. [5,25]
Brimstone	Null	6 + 8*	2 + 12	Liu et al. [32] and Jang et al. [5,25]
Cheyenne	2*	7 + 9	5 + 10	Dupont et al. [33] and Jang et al. [5,25]
Chinese Spring	Null	7 + 8	2 + 12	Liu et al. [32] and Jang et al. [5,25]
Clement	Null	6 + 8*	2 + 12	Liu et al. [32] and Jang et al. [5,25]
Dajoong	2*	13 + 16	2.2 + 12	Jang et al. [5,25]
Glenlea	2*	7^OE^ + 8*	5 + 10	Naeem and Sapirstein [34] and Jang et al. [5,25]
Gobun	Null	7 + 9	2 + 12	Jang et al. [5,25]
Hanno	1	14 + 15	5 + 10	Gao et al. [22] and Jang et al. [5]
Imbros	1	14 + 15	2 + 12	Kazman et al. [35] and Jang et al. [5]
Insignia	1	20 + 20	5 + 10	Branlard et al. [36] and Jang et al. [5,25]
IT166460	1	7^OE^ + 8*	2 + 12	Cho et al. [37] and Jang et al. [5,25]
Joongmo 2008	Null	17 + 18	5 + 10	Jang et al. [5,25]
Manital	2*	17 + 18	2 + 12	Liu et al. [32] and Jang et al. [5]
Nanbu-komugi	1	7 + 8	4 + 12	Liu et al. [32] and Jang et al. [5,25]

**Table 2:** Reproducibility of retention times for 22 HMW-GS determined by RP-UPLC in 16 standards.

Type	HMW-GS	Retention Time (min) ^1^	RSD (%) ^2^	Number of Cultivars ^3^
Ax	1	5.37 ± 0.01	0.25	5
	2*	5.24 ± 0.01	0.22	5
Bx	6	4.56 ± 0.01	0.19	2
	7	4.67 ± 0.02	0.42	5
	7^OE^	4.63 ± 0.02	0.35	2
	13	4.23 ± 0.01	0.10	2
	14	4.67 ± 0.01	0.16	2
	17	4.71 ± 0.01	0.27	2
	20x	5.09 ± 0.01	0.11	1
By	8	4.11 ± 0.01	0.24	2
	8*	3.74 ± 0.01	0.31	4
	9	3.75 ± 0.01	0.12	3
	15	3.72 ± 0.01	0.17	2
	16	3.96 ± 0.01	0.14	2
	18	3.73 ± 0.01	0.23	2
	20y	3.70 ± 0.01	0.07	1
Dx	2	4.44 ± 0.01	0.26	8
	2.2	4.31 ± 0.01	0.11	2
	4	4.48 ± 0.01	0.12	1
	5	4.34 ± 0.01	0.32	5
Dy	10	3.00 ± 0.01	0.39	5
	12	2.97 ± 0.01	0.33	11

^1^ Average retention times determined from 10 analyses per cultivar. ^2^ Relative standard deviation. ^3^ 16 standard wheat cultivars listed in Table 1.

**Table 3:** Allelic frequency of HMW-GS inferred by the optimized method in 1787 wheat germplasm.

Locus	HMW-GS	Allele *	Number of Cultivars	Frequency (%)
*Glu-A1*	1	*Glu-A1a*	418	23.39
	2*	*Glu-A1b*	774	43.31
	Null	*Glu-A1c*	595	33.30
*Glu-B1*	6	*Glu-B1-1d*	8	0.45
	6 + 8*	*Glu-B1ca*	65	3.64
	7	*Glu-B1a*	64	3.58
	7 + 8	*Glu-B1b*	473	26.47
	7 + 8*	*Glu-B1ce*	219	12.26
	7 + 9	*Glu-B1c*	503	28.15
	7^OE^ + 8*	*Glu-B1al*	33	1.85
	7^OE^ + 9	unknown	2	0.11
	8	*Glu-B1aj*	1	0.06
	8*	*Glu-B1-2o*	1	0.06
	9	*Glu-B1-2b*	1	0.06
	13 + 16	*Glu-B1f*	67	3.75
	14 + 15	*Glu-B1h*	2	0.11
	15	*Glu-B1-2e*	7	0.39
	17 + 18	*Glu-B1i*	219	12.26
	20y	*Glu-B1-2z*	2	0.11
	20 + 20	*Glu-B1e*	120	6.72
*Glu-D1*	2 + 12	*Glu-D1a*	758	42.42
	2	*Glu-D1k*	1	0.06
	2.2 + 12	*Glu-D1f*	150	8.39
	4 + 12	*Glu-D1c*	38	2.13
	5	*Glu-D1-1d*	2	0.11
	5 + 10	*Glu-D1d*	837	46.84
	12	*Glu-D1l*	1	0.06

* Gene nomenclature system based on Catalogue of gene Symbols for Wheat.

## Data Availability

The data presented in this study are available in supplementary material.

## Supplementary Materials

The following are available online. Figure S1: RP-UPLC chromatograms of the glutenin fraction in Chinese Spring using (a) ACQUITY Peptide BEH C_18_ column and (b) ACQUITY Protein BEH C_4_ column eluted with the same linear ACN gradient of 23–41% for 30 min; Figure S2: Overlap of HMW-GS chromatograms run 10 times using the optimized RP-UPLC method from the Chinese Spring; Figure S3: SDS-PAGE analysis of HMW-GS in Glenlea, Cheyenne and Hanno. Two group of HMW-GS, which are indistinguishable by RP-UPLC due to overlap retention time, are underlined in blue (1Bx7/1Bx7^OE^ and 1Bx14) and red (1By8*, 1By9 and 1By15); Figure S4: PCR amplification to determine the 1Bx7^OE^ genotype. Left and Right refer to the junction of the LTR retrotransposon associated with 1Bx7^OE^ [17]. The left and right junction of the retroelement and the duplicated region generated 447-bp and 844-bp amplicons respectively in 1Bx7^OE^. Cheyenne (CH) and Chinese Spring (CS) were used as negative controls for 1Bx7^OE^. Glenlea (GL) and IT166460 (IT) were used as positive controls for 1Bx7^OE^ [41]. Marker (M) is 100bp Plus DNA Ladder; Table S1: Reproducibility of HMW-GS analysis in Chinese Spring by RP-UPLC; Figure S5: RP-UPLC chromatograms of the HMW-GS in (a) Insignia (b) Manital and (c) mixture of Insignia and Manital; Table S2: HMW-GS compositions identified by the optimized method in 1787 wheat germplasm. Wheat accessions harboring HMW-GS allelic combination associated with good bread-making quality and unique null subunits are marked in the note; Table S3: Frequency of HMW-GS allelic combination in 1787 wheat germplasm. HMW-GS allelic combinations associated with good bread-making quality are indicated in the note.

## Sample Availability

Samples of the compounds are not available from the authors.
