# Supplementary material for: A Rapid, Reliable RP-UPLC Method for Large-Scale Analysis of Wheat HMW-GS Alleles
Source: Molecules. 2021 Oct 13;26(20):6174. doi: 10.3390/molecules26206174 (PMC8540670; doi:10.3390/molecules26206174)
Supplement: Supplementary file 1 [file molecules-26-06174-s001.zip › molecules-1382736-supplementary.pptx]

## Slide 1
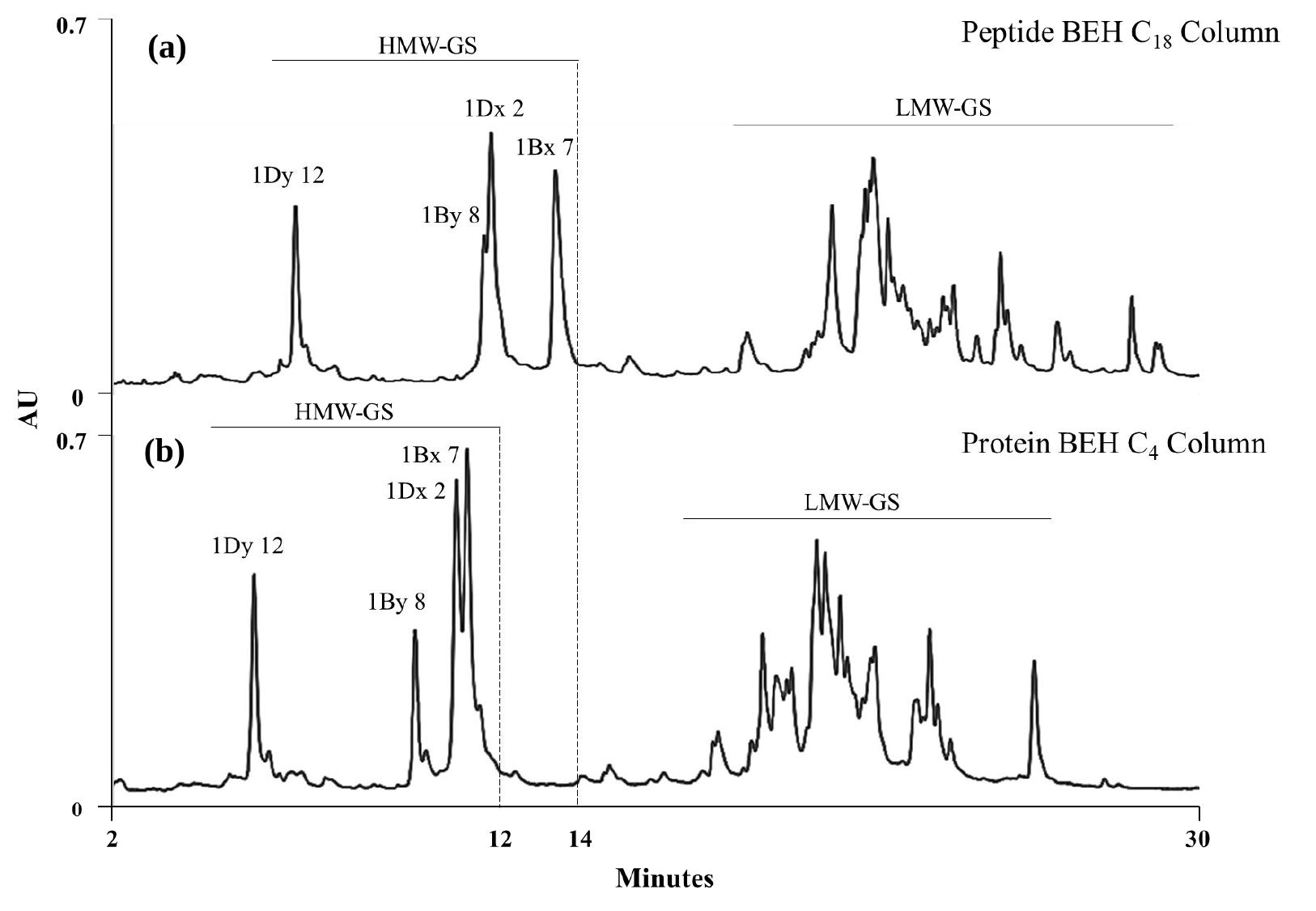

(a)
(b)

## Slide 2
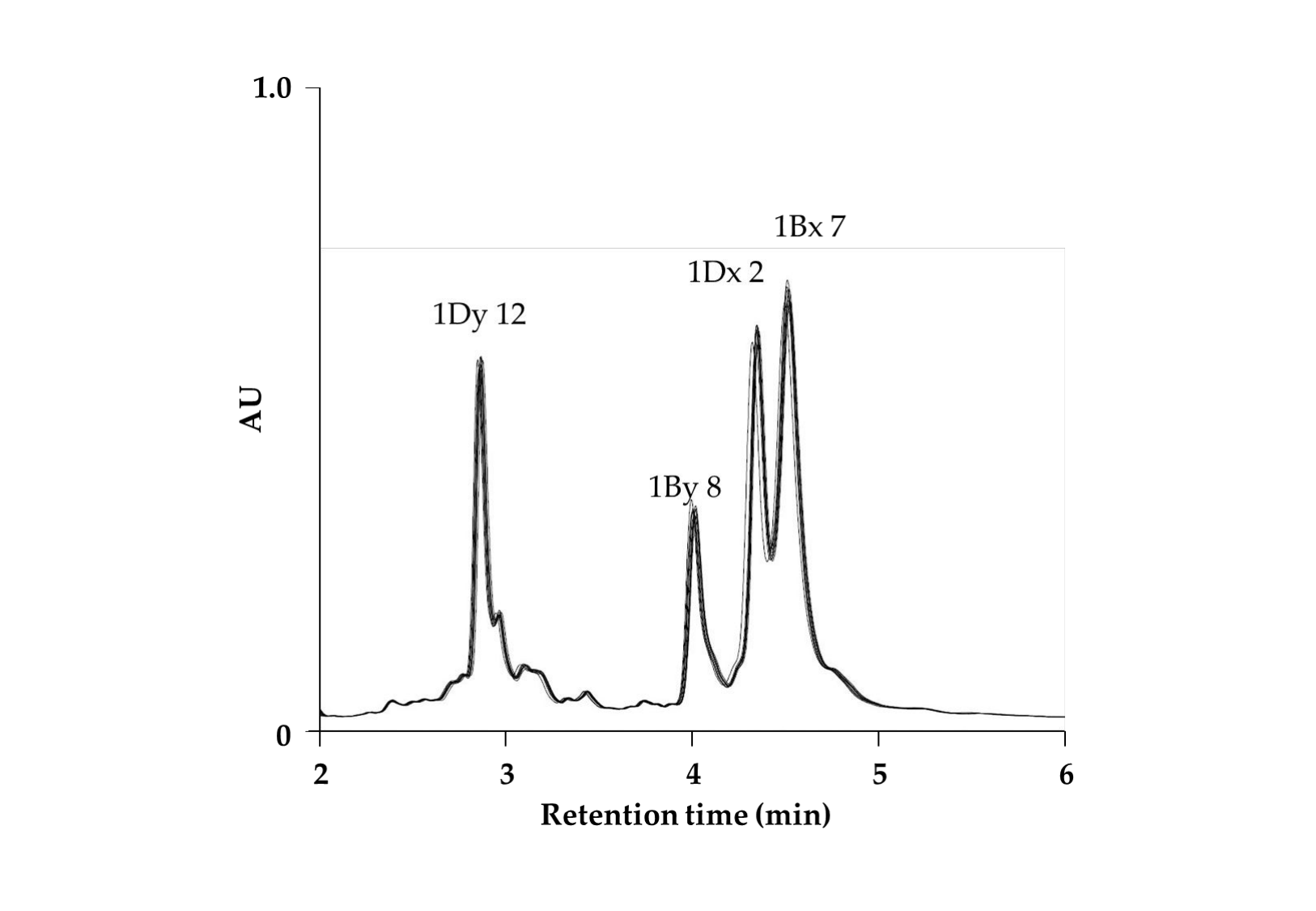

## Slide 3
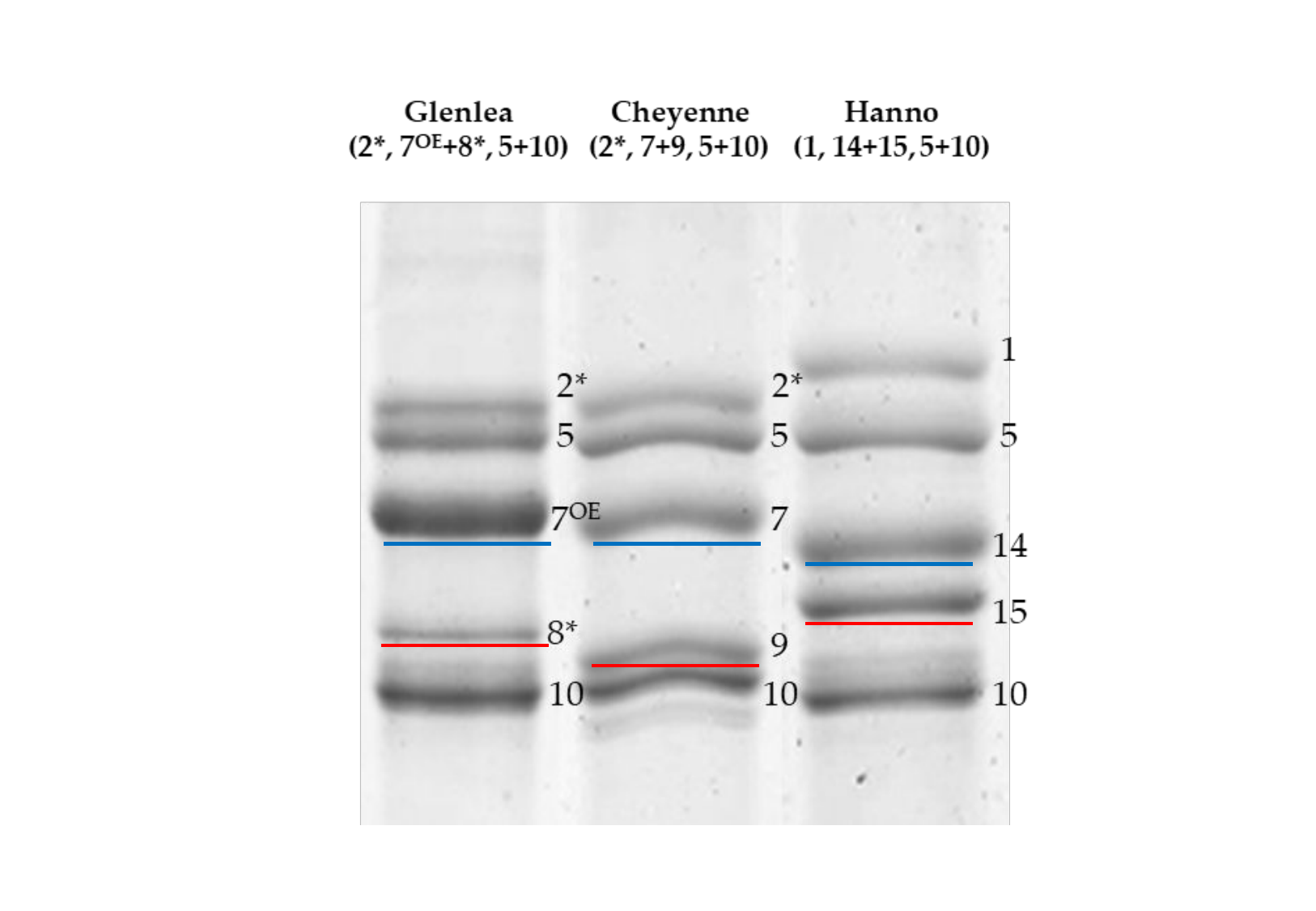

## Slide 4
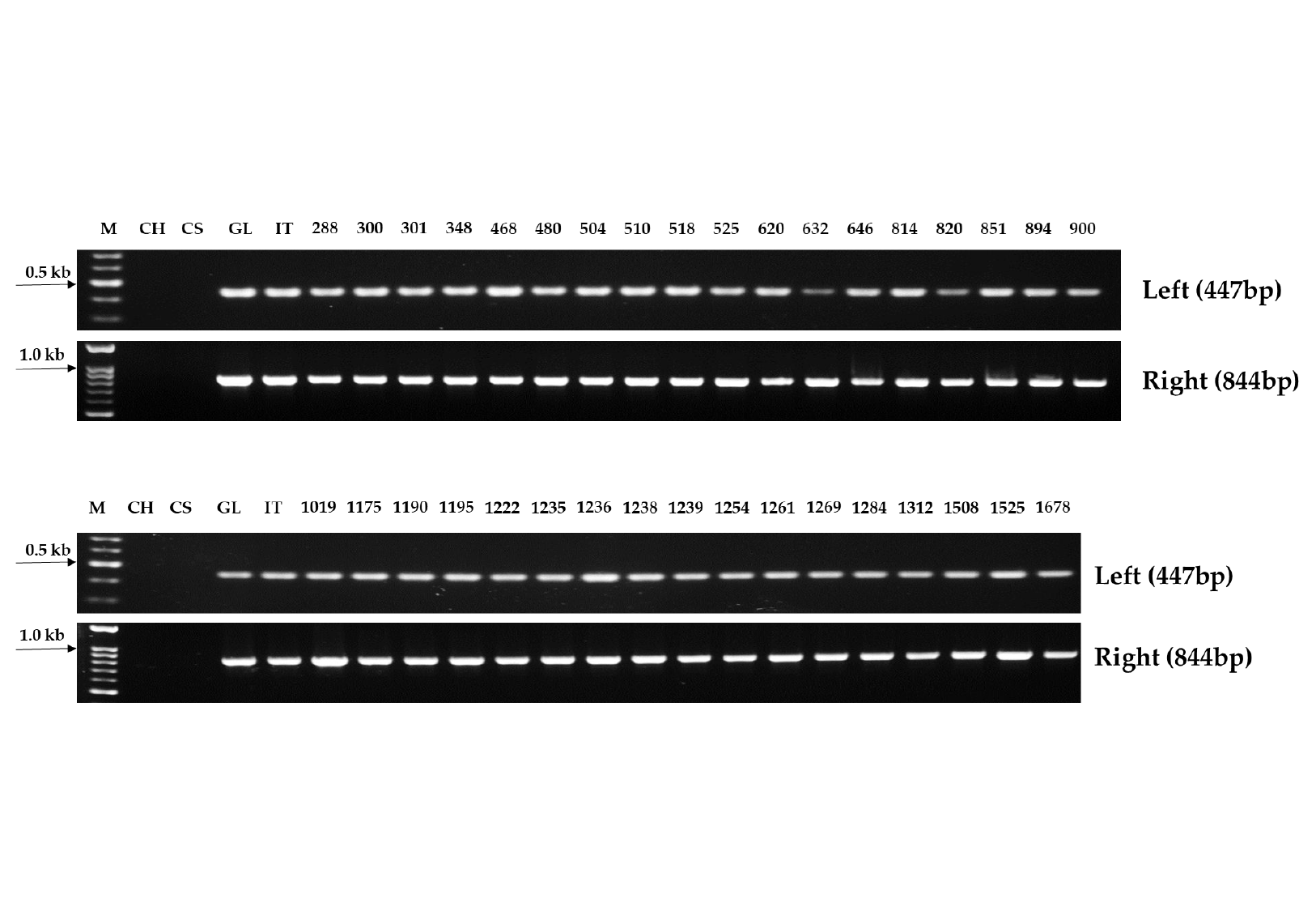

## Slide 5
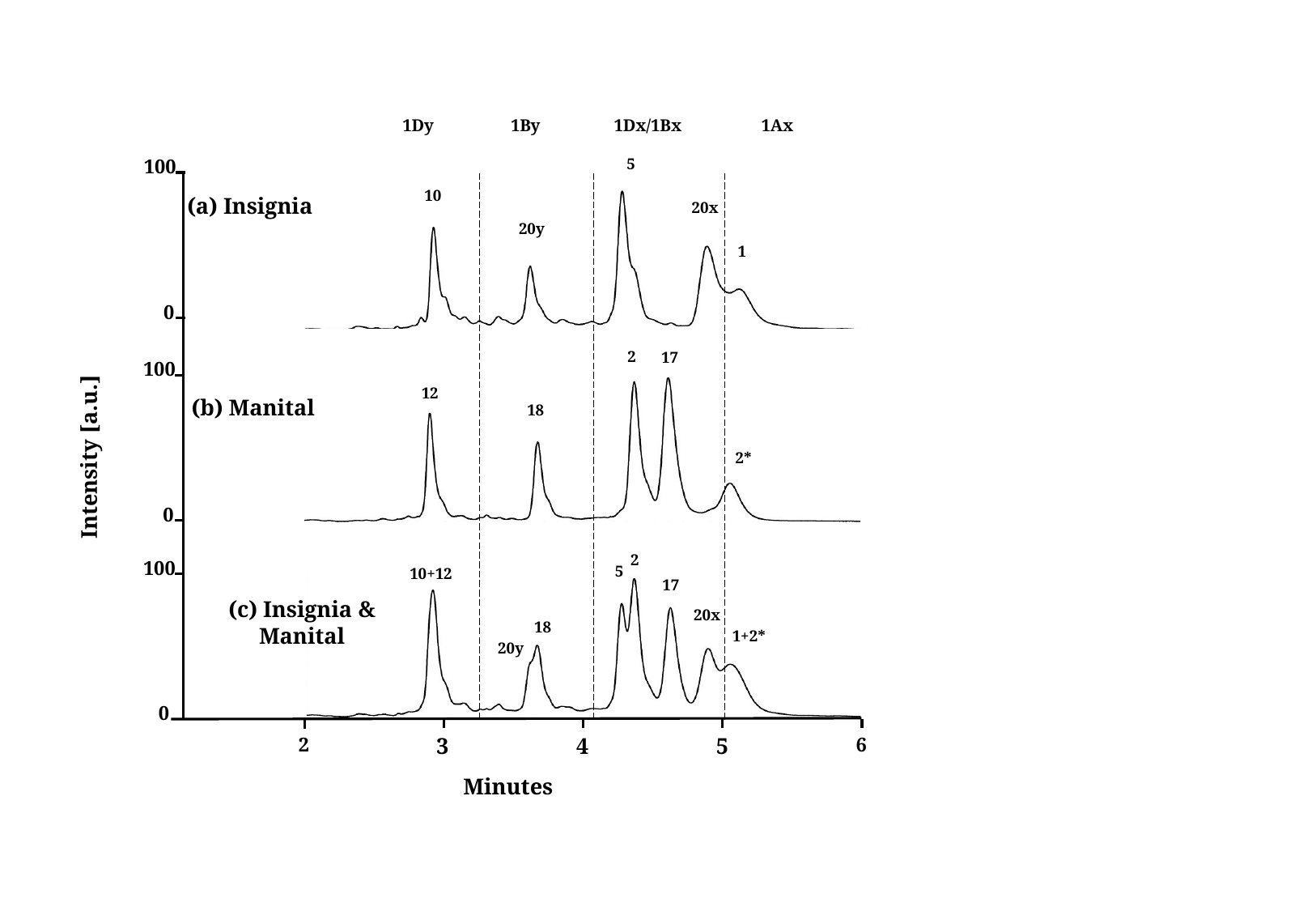

1Dy
1By
1Dx/1Bx
1Ax
5
100
10
(a) Insignia
20x
20y
1
0
Intensity [a.u.]
2
17
100
12
(b) Manital
18
2*
0
2
100
5
10+12
17
(c) Insignia & Manital
20x
18
1+2*
20y
0
2
3
4
5
6
Minutes
